# Self-Perceptions of Aging Predict Recovery After a Fall: Prospective Analysis From the English Longitudinal Study of Aging

**DOI:** 10.1111/jgs.19486

**Published:** 2025-05-21

**Authors:** Mathew W. Hill, Elmar Kal, Stephen Ronald Lord, Hayley Wright, David Broom, Toby J. Ellmers

**Affiliations:** 1 Centre for Physical Activity, Sport and Exercise Sciences, Coventry University, Coventry, UK; 2 Centre for Cognitive Neuroscience, Department of Health Sciences, College of Health, Medicine, and Life Sciences, Brunel University London, London, UK; 3 Falls, Balance and Injury Research Centre, Neuroscience Research Australia, Randwick NSW 2031, University of New South Wales, Sydney, Australia; 4 Centre for Intelligent Healthcare, Coventry University, Coventry, UK; 5 Department of Brain Sciences, Imperial College London, London, UK

**Keywords:** falls, physical function, recovery, self-perceptions of aging

## Abstract

**Objective:**

To investigate how mindsets around aging at baseline affect physical recovery following a subsequent fall.

**Design:**

Longitudinal observational study.

**Setting:**

English Longitudinal Study of Aging (ELSA).

**Participants:**

We analyzed data for 694 individuals who had not fallen in the 2 years prior to baseline (Wave 4) but experienced a fall during follow-up (between Waves 4 and 5).

**Measurements:**

Self-perceptions of aging at baseline (Wave 4) and gait speed, activities of daily living (ADL) dependence, and physical (in)activity after a fall at a 2-year follow-up (Wave 5). Multivariable logistic regression analyses were used to determine to what extent aging-related mindset variables as measured at baseline predicted outcome measures at follow-up.

**Results:**

In a fully-adjusted model controlling for confounding baseline factors (including baseline gait speed, ADL dependence and physical inactivity), individuals with positive self-perceptions of aging at baseline had significantly lower odds of slow gait speed (OR = 0.729; 95% CI = 0.627–0.849), ADL dependence (OR = 0.667; 95% CI = 0.561–0.792) and physical inactivity (OR = 0.795; 95% CI = 0.700–0.904) following a fall at a 2-year follow-up.

**Conclusions:**

These findings identify self-perceptions of aging as a strong predictor of physical recovery and disability following a fall, independent of other important factors such as age, gender, and pre-fall physical function. These novel observations advance our understanding of the psychological factors impacting physical recovery from a fall. Future work should explore if targeting such perceptions can directly improve physical recovery and outcomes following a fall.

## 1 Introduction

Falls in older adults are a major public health concern, leading to high levels of physical disability and institutionalization [1, 2]. Yet not every older adult who falls will experience subsequent declines in physical function [3, 4]. Enhancing our understanding of the factors that mediate this relationship is crucial for developing interventions to prevent physical disability following a fall. While baseline physical function and the seriousness of the fall have been shown to be important predictors of this relationship [2–4], less is known about moderating psychological factors such as expectations and attitudes. This reflects a critical gap in knowledge given that successful aging is now widely considered to be as much a psycho-social process as a physiological one [5–8].

Studies have identified mindsets (assumptions that an individual holds about themselves and the world) as a key predictor of physical function in later life [8]. For instance, self-perceptions of aging (i.e., beliefs, attitudes, and assumptions about aging and their own aging process) have been shown to independently predict generalized functional health over an 18-year period [9]. Similarly, in a longitudinal cohort study, greater optimism (i.e., the belief that the future aging process will be favorable) about aging independently predicted reduced risk of stroke over 6 years of follow-up [10]. More specific to physical disability, Levy et al. [11] found that older adults with positive self-perceptions of aging experienced slower rates of decline in ability to perform activities of daily living (ADLs) over a ~10-year period. Individuals with more positive self-perceptions were also nearly 50% more likely to fully recover from severe disability in ADLs. Particularly relevant to the domain of fall prevention, older adults acutely exposed to positive verbal descriptions about aging performed significantly better on a subsequent clinical balance task [12]. These collective findings have been interpreted with respect to the “stereotype embodiment theory” [7]. This theory explains how negative perceptions about aging can become a self-fulfilling prophecy, with these individuals less likely to engage in preventive health behaviors due to viewing health problems as an inevitable and uncontrollable consequence of aging [13].

Despite the wealth of information linking mindsets around aging to various negative health outcomes in later life, little is known about how such mindsets affect physical recovery following a fall. One study has reported an association between optimism and physical health after a fall [14]. However, as this study assessed generalized optimism (e.g., “In uncertain times, I usually expect the best”) rather than optimism specific to aging—with optimism also assessed after the fall had occurred—it is difficult to draw definitive and directional conclusions. For instance, perhaps these individuals are now simply less optimistic due to the fall having had a larger impact on their physical health, rather than optimism affecting the recovery itself. The present work therefore adopts a longitudinal prospective design to investigate how mindsets specifically around aging (at baseline) affect physical recovery following a subsequent fall. We focused our investigation on new fallers to minimize the likelihood that participants have pre-existing fall-related experiences that could confound the relationship between aging mindsets and fall recovery outcomes. We hypothesized that positive mindsets around aging—specifically, greater optimism and self-perceptions about the aging process, and lower subjective (or, “psychological”) age—would be protective against negative physical consequences following a fall.

## 2 Materials and Methods

### 2.1 Data Source

This study used data from the English Longitudinal Study of Aging (ELSA), a cohort study of a representative sample of community-dwelling men and women aged 50 years and older living in England. The first wave of data was conducted in 2002/03 (Wave 1), with subsequent waves comprising face-to-face interviews and self-report questionnaires conducted every 24 months. The ELSA data and general methods of data collection are detailed at www.elsa-project.ac.uk. For the present study, data were gathered at Wave 4 (baseline) and Wave 5 (follow-up). Data collected at Wave 4 were used as the baseline for this analysis because it was the first wave assessing all baseline parameters needed for our investigation. ELSA received ethical approval from the National Hospital for Neurology and Neurosurgery & Institute of Neurology Joint Research Ethics Committee (Wave 4; 07/H0716/48) and the Berkshire Research Ethics Committee (Wave 5; 09/H0505/124). All participants gave full informed consent.

### 2.2 Study Population: First Time Fallers

**Figure 1:**
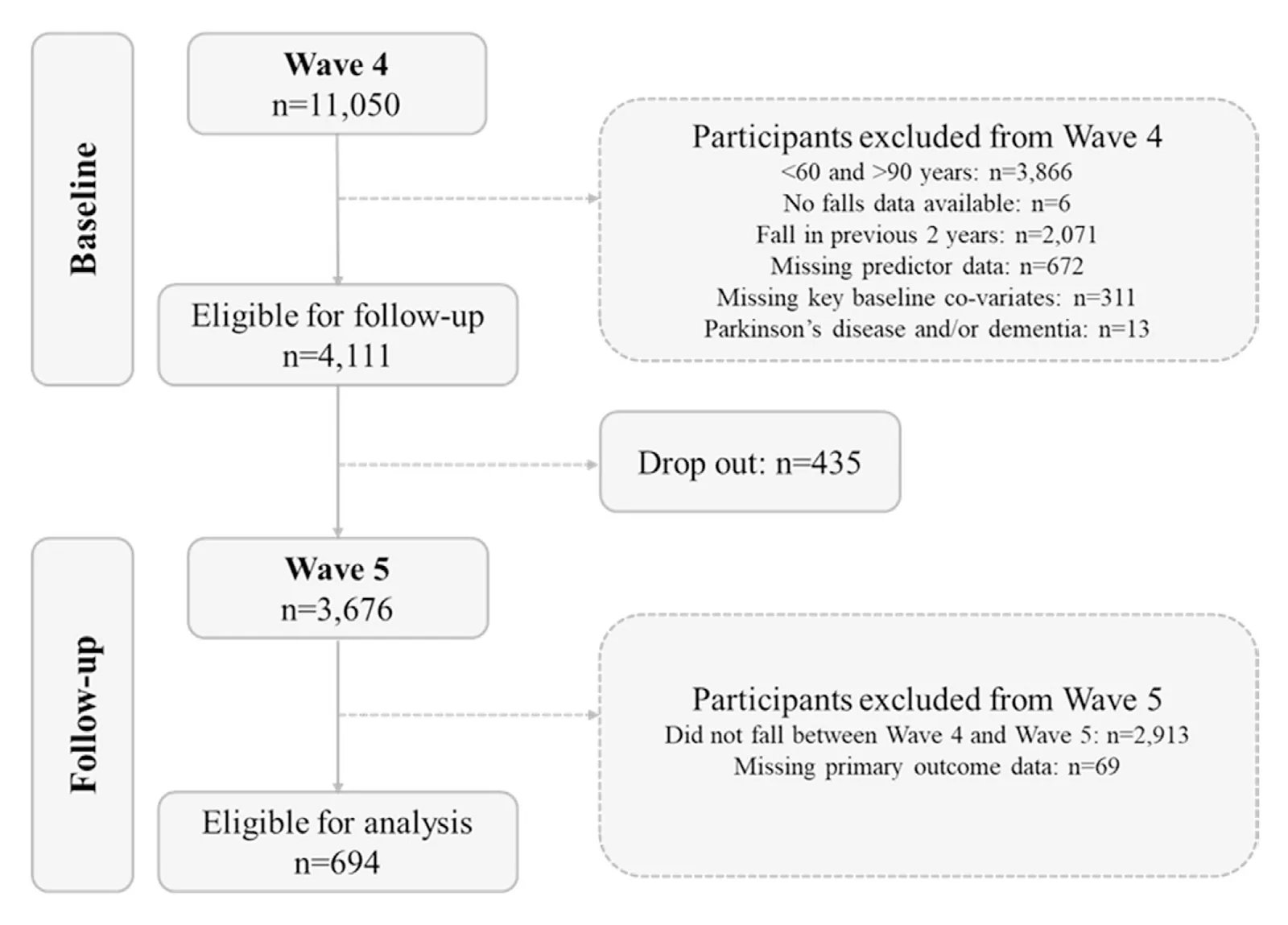
Cohort flow chart of participants included in the analysis. As we were interested in “new fallers”, we excluded participants who had fallen in the 2 years prior to baseline (Wave 4), and also participants who did not fall during follow-up (i.e., between Waves 4 and 5).

At Wave 4 (2008/09) the sample consisted of 11,050 individuals. Participants were excluded if they were aged < 60 or > 90 years (*n* = 3866) or were diagnosed with either Parkinson's disease, Stroke or Dementia (*n* = 13). As we were interested in outcomes following a “first” fall, participants were excluded if they reported any falls (defined as “whether fallen down”) in the 24 months prior to Wave 4 (*n* = 2071) or if no fall data for this period were recorded (*n* = 6). The occurrence of falls in the previous 24 months were assessed at each follow-up visit. A further 983 participants were excluded due to missing data for either the key predictor variables (*n* = 672) or key baseline covariates (*n* = 311). The sample with complete Wave 4 data comprised 4111 participants. Of these, 435 participants did not complete the Wave 5 assessments. As we were interested in physical recovery following a fall, we then also excluded participants who (i) did not experience a fall between Wave 4 and 5 (*n* = 2913), and (ii) had missing data for our Wave 5 primary outcome variables (*n* = 69). Although the exact timing of each fall within this period was not recorded, we classified these incidents as occurring at some point between the two waves, based on participant self-reports and any available follow-up data. The final sample for this analysis comprised 694 participants (mean ± SD age; 69.8 ± 7.2 years, female; *n* = 404 [58%]) (Figure 1).

### 2.3 Predictor Variables: Mindsets Around Aging

Mindsets, broadly defined, refer to the assumptions and beliefs we hold that shape how we see the *world* and *ourselves*. For this study, we chose to focus specifically on *self-perceptions* of aging and *optimism* about aging due to their demonstrated relevance in prior research on health outcomes, such as general health in later life [9] and physical recovery from disability [11]. As per previous research [10], *self-perceptions of aging* were assessed with the following two items CASP19 quality of life questionnaire [15]: (1) “My age prevents me from doing the things I would like to do” and (2) “My health stops me from doing the things I want to do”. *Optimism about aging*, also from the CASP19, were assessed with the following items: (1) “I feel that life is full of opportunities” and (2) “I feel that the future looks good for me”. Each statement was assessed on a four-point Likert scale ((1) “often”, (2) “sometimes” (3) “not often” or (4) “never”) as to the extent to which the description applies to the participants feelings about their life. We reverse coded the *optimism* responses so that for both outcomes higher values indicate more positive views about aging. Summed scores (range 2–8) were calculated for *optimism* and *self-perceptions of aging*. We additionally included *subjective age* as it provides insight into how individuals internalize their aging process in real-time, distinct from broader attitudes and expectations like self-perceptions and optimism about aging. This perspective is essential for capturing the multifaceted influence of aging mindsets on recovery outcomes. *Subjective age* was assessed by asking participants to specify, in years, how old they felt. In-line with previous work, a “proportional discrepancy” score was calculated by subtracting participants' subjective age from their chronological age, and these difference scores were divided by chronological age [16]. Positive values indicate that participants feel younger than their chronological age (e.g., a score of 0.2 indicates that participants felt 20% younger than their actual age).

### 2.4 Primary Outcomes: Physical Function and Disability

Our primary outcomes were gait speed, ADL dependence, and physical inactivity at Wave 5 follow-up (following a fall). As a measure of generalized physical function, participants were asked to walk 2.4 m at their usual pace. The times of the two trials were recorded using a stopwatch, and the mean time was calculated. Gait speed was calculated by dividing distance (in meters) by mean time (in seconds). Based on recommendations from the World Guidelines for Falls Prevention and Management [17], we stratified participants into a “slow” gait speed category using the cut-off value of < 0.8 m/s. As a measure of physical disability, participants reported being dependent on others to perform basic ADLs [18]. Basic ADLs consisted of six activities: (1) dressing, (2) walking across a room, (3) bathing or showering, (4) eating, (5) getting in or out of bed, and (6) using the toilet. ADL dependence was defined as having one or more ADL limitations. Physical *inactivity* was assessed by asking participants about the frequency in which they engaged in vigorous, moderate, and mild physical activity. For each level of activity, the response options were: (1) *more than once per week*, (2) *once per week*, (3) *1–3 times per month*, and (4) *hardly ever*. Responses were subsequently categorized as *Active* (moderate or vigorous activity more than once a week) or *Insufficiently Active* (physical activity once a week or less or undertaking mild activities only), based on physical activity recommendations and previous research [19].

### 2.5 Covariates

Covariates included in this analysis included the following variables collected at baseline (Wave 4): gait speed, ADL dependence, physical activity levels, age (in years), gender (male/female), self-reported general health (dichotomised into good/excellent or very poor/poor) and depressive symptoms (evaluated using the 8-item version of Centre for Epidemiologic Studies Depression Scale (CES-D) with a score of ≥ 4 indicating higher symptoms and possible depression) [20]. In addition, whether the fall/s experienced between Wave 4 and 5 resulted in physical injury (defined as “whether injured seriously enough to need medical treatment”) was also controlled for in the analyses.

### 2.6 Statistical Analysis

We conducted three separate multivariable logistic regression analyses (performed using SPSS version 29.0) to analyze to what extent the three aging related mindset variables as measured at baseline predicted reduced physical function (gait speed < 0.80 m/s), physical inactivity, and ADL-dependence at follow-up. Each analysis was conducted in two steps. In the first step, we included only the aging-related mindset variables, to obtain unadjusted odds ratios for each outcome. Second, we added the following control variables (as collected at baseline) to obtain adjusted odds ratios: Age in years, gender, depression (CES-D), self-reported health (dichotomised into good to excellent or very poor to poor, respectively), injurious falls between baseline and follow-up, gait speed (cm/s), physical inactivity, and ADL-dependence. Model fit was evaluated using Nagelkerke R2. There were no multicollinearity issues (*r*'s ≤ 0.418; variance inflation factors ≤ 1.6, tolerances ≥ 0.6). The assumption of linearity for continuous variables was met for all analyses (as evidenced by the absence of significant “predictor * ln(predictor)” interactions indicating a linear relationship between predictor and outcome). Alpha was set at 0.05 for all analyses.

## 3 Results

Detailed characteristics of the participants are presented in Table 1. In all, at baseline (Wave 4) participants' gait speed was relatively low, but on average above the cut-off for “slow” gait speed (0.80 m/s). Also, a substantial minority of participants were ADL dependent (17%) and physically inactive (32%) (Table 1).

### 3.1 Slow Gait Speed at Follow-Up

In total, 260 individuals (37%) were classified as having “slow gait speed” at follow up. In an unadjusted regression model including aging-related mindsets only, “self-perceptions of aging” were a significant predictor of slow gait speed status at follow-up (indicative of poor physical function; OR: 0.575 [0.513, 0.645]). While attenuated, self-perceptions of aging remained a significant predictor even when controlling for other relevant variables (OR: 0.729 [0.627, 0.849]). This suggests that each 1-point improvement in self-perceptions of aging is associated with ~27% reduced likelihood of having slow gait speed at follow-up. Of the control variables, higher age (1.074 [1.041, 1.109]) and physical inactivity (1.599 [1.008, 2.538]) at baseline significantly predicted slow gait speed status at follow-up, while higher gait speed at baseline was associated with lower odds of slow gait speed at follow-up (0.088 [0.057, 0.135]) (Table 2).

### 3.2 ADL Dependence at Follow-Up

At follow-up, 130 individuals (19%) reported being dependent on others for at least one ADL activity. The unadjusted regression model revealed that “self-perceptions of aging” (0.518 [0.447, 0.601]) significantly predicted ADL dependence at follow-up. Again, while attenuated, this factor remained a significant predictor when controlling for other key variables (0.667 [0.561, 0.792]). Hence, each 1-point improvement in self-perceptions of aging was associated with an ~33% reduction in likelihood of being physically inactive at follow-up. Of the control variables, both ADL dependence status (5.249 [3.130, 8.801]) and worse self-reported health at baseline (2.752 [1.459, 5.189]) were associated with greater odds of ADL-dependence at follow-up, while higher gait speed at baseline was associated with lower odds of ADL dependence at follow-up (0.386 [0.231, 0.644]) (Table 3).

### 3.3 Physical Inactivity at Follow-Up

At follow-up, 275 individuals (40%) were classified as being physically inactive in daily life. The unadjusted regression model revealed that both “optimism” (OR: 0.809 [0.719, 0.909]) and “self-perceptions of aging” (OR: 0.676 [0.609, 0.751]) significantly predicted inactivity status at follow-up. However, only “self-perceptions of aging” remained significant when controlling for other key variables (0.795 [0.700, 0.904]). Hence, each 1-point improvement in self-perceptions of aging was associated with an ~20% reduction in likelihood of being physically inactive at follow-up. Of the control variables, being physically inactive at baseline was significantly associated with increased odds of physical inactivity at follow-up (3.454 [2.350, 5.077]), while higher gait speed at baseline was associated with lower odds of physical inactivity at follow-up (0.492 [0.335, 0.720]) (Table 4).

### 3.4 Moderating Effects of Gender, Age Group, and Injury Status

Additional exploratory analyses examining the potential moderating effects of age, gender, and injurious falls on the relationships between self-perception of aging and gait speed (all *p*s > 0.152), physical activity (all *p*s > 0.055), and ADL independence (all *p*s > 0.076) revealed no significant interaction effects.

## 4 Discussion

This study provides the first insight into associations between mindsets around aging at baseline and subsequent physical function and disability following a future fall. In support of our hypothesis, we found that older individuals who had more positive self-perceptions of aging at baseline were better protected against negative physical consequences following a fall. Specifically, they had significantly lower odds of slow gait speed, ADL dependence and physical inactivity after a fall that occurred over an ensuing 2-year period. These results remained statistically significant after adjusting for important covariates including baseline gait speed, ADL dependence and physical inactivity, suggesting that self-perceptions of aging may be an important factor associated with physical recovery following a fall, independent of other important covariates such as age, gender, and pre-fall physical function. Contrary to our hypotheses, these predictive associations were restricted to self-perceptions of aging—with results identifying a limited role for optimism about aging and subjective age. In line with previous work, we interpret these findings with respect to the “stereotype embodiment theory” [7], whereby negative perceptions about aging discourage health seeking behaviors due to poor health being viewed as an inevitable and uncontrollable consequence of aging [13]. Specifically, we propose that individuals with more positive self-perceptions of aging may experience higher levels of self-efficacy [21], which could reduce fear of falling [22] and promote greater engagement in physical activity [23]. Those with positive self-perceptions of aging may also be more likely to maintain social connections [24] and adhere to rehabilitation [25], contributing to better recovery outcomes.

Our findings add to the growing body of evidence illustrating the potential critical influence that self-perceptions of aging exert across physical function in later life. Self-perceptions of aging can be defined as an individual's expectation and attitude towards the aging process. Previous longitudinal evidence has shown that holding a negative self-perception of aging robustly predicts declining physical function [11, 26–28], increased ADL disability [8, 29], greater frailty [30, 31] and reduced physical activity [32] in later life. However, the present study is the first to use a longitudinal prospective cohort design to explore how mindsets about aging predict physical function following the occurrence of a fall. The ORs imply that following a fall, an individual scoring the maximum points on our self-perceptions of aging measure would have 162% (95% CI = 91%–224%) lower odds of exhibiting slow gait speed, 200% (95% CI = 125%–264%) lower odds of ADL dependence, and 123% (95% CI = 58%–180%) lower odds of physical inactivity compared to an individual scoring the lowest possible points. These results identify self-perceptions of aging may be an independent factor associated with physical recovery from a fall—highlighting the protective effects that positive self-perceptions can exert following a fall.

Each of the outcomes (slow gait speed, ADL dependence, physical inactivity) examined in the present study has been shown to predict falls [17, 33–37]. Indeed, the 2022 World Falls Guidelines [17] used slow gait speed (using the same stratification cut-off value as the present study) as one of the core risk factors for determining if an older adult is deemed to be at a high risk for falls. Previous randomized controlled trials have shown that negative self-perceptions of aging are modifiable [38–40]. Future work should therefore explore if targeting such perceptions can directly improve physical recovery and outcomes following a fall. If so, this would further emphasize the importance of clinically assessing self-perceptions of aging using, for instance, the two-item assessment used in the present study (given its low clinical burden).

Contrary to our hypothesis, our findings revealed limited evidence for the role of optimism about aging in influencing physical recovery from a fall. In an initial unadjusted analysis, having a more optimistic mindset about aging was associated with greater physical activity levels following a fall over the follow-up period, though this association did not survive adjustment for other confounding factors (OR = 0.880 [95% CI = 0.774, 1.000], *p* = 0.050). Our findings contrast with those of Ruthig et al. [14] who found that generalized optimism was linked to better physical health after a fall. However, this discrepancy is likely explained through differences in study design (longitudinal in the present work vs. cross-sectional assessed previously) and in the conceptualization and measurement of optimism between studies (specific optimism about aging in the present work vs. generalized optimism assessed previously). One possible reason for why self-perceptions of, but not optimism about, aging predicted recovery from a fall could be that self-perceptions are more directly aligned to individuals' beliefs about their current physical state. In contrast, optimism about aging reflects a broader, future-oriented perspective that, while beneficial for general well-being, may not necessarily drive the specific, immediate behaviors required for physical recovery after a fall.

### 4.1 Study Limitations

Although we adopted a longitudinal prospective design controlling for important covarying factors, there are several limitations to our study. Most importantly, self-perceptions and optimism about aging were determined through four items selected from the wider CASP19 questionnaire. Although similar approaches have been employed in previous research (see [10]), assessing mindsets about aging through only four questions is likely an oversimplification of a complex and multifaceted reality [22]. Although the short nature of these assessments increases the potential utility in clinical practice, we acknowledge our findings need to be replicated using more thorough assessments of self-perceptions and mindsets around aging (e.g., Attitude Towards Own Aging subscale, and the Aging-related Cognitions scales [41, 42]). Further limitations relate to the non-standard definition of a fall within the ELSA assessment [43], as older adults can interpret the meaning of a fall in many ways [44]. This issue might be further complicated because individuals with a negative perception of aging may differ in their recall of negative events [29]. Although we controlled for the severity of the fall experienced, we were unable to control for the time between the occurrence of the fall (during follow-up) and the assessment of the follow-up outcomes. While we consider it unlikely that the timing of the fall would systematically differ based on self-perceptions of aging—and thus unlikely to have introduced significant bias—future research could examine the effects of the timing of a fall on falls recovery outcomes. Finally, due to the design (in which self-perceptions were assessed only at baseline, and every participant included experienced a subsequent fall), we were unable to examine possible reciprocal relationships between self-perceptions of aging and falls. Future work should explore this relationship to better understand how self-perceptions of aging relate to falls and associated consequences.

### 4.2 Conclusions and Future Research Questions

The present findings identify self-perceptions of aging as a robust predictor of physical function and disability following a fall, independent of other important factors such as age, gender, and pre-fall physical function. These novel observations advance our understanding of the psychological factors impacting physical recovery from a fall and suggest that assessment of self-perceptions of aging could help identify older adults requiring additional support following a fall. Future work should seek to explore both the reciprocal relationship between falls and self-perceptions of aging, as well as the clinical utility of interventions designed to target self-perceptions of aging on adverse fall-related outcomes. We also recommend that future studies consider broader factors influencing falls and recovery following a fall, such as socioeconomic (wealth, education and isolation) status.

## Figures and Tables

**Table 1:** Characteristics for participant group (*N* = 694).

	Mean ± SD (range)^a^
General	
Age in years	69.8 ± 7.2 (60–89)
Gender (female; *N* (%))	404 (58%)
Ethnicity (white; *N* (%))	687 (99%)
Self-reported health (1–5, higher = better self-reported health, median ± IQR (range))	3 ± 1 (1–5)
ADL-dependence (yes; *N* (%))^b^	121 (17%)
CES-D (0 –8, higher = more depressive symptoms)	5.0 ± 1.4 (0–8)
Physical function	
Gait speed (lower values indicate slower walking speed) (m/s)	0.89 ± 0.29 (0.11–2.04)
Injurious fall between baseline and follow-up (*N* (%))^c^	165 (24%)
Physically inactive (*N* (%))^d^	224 (32%)
Aging mindsets	
Optimism about aging (2–8, higher = greater optimism)	6.1 ± 1.5 (2–8)
Self-perceptions about aging (2–8, higher = greater self-perception)	5.3 ± 1.7 (2–8)
Subjective age^e^	0.21 ± 0.25 (–0.61–1.13)

*Note:* Baseline data for baseline (Wave 4) unless otherwise specified. Abbreviations: ADL, activities of daily living; CES-D, Centre for epidemiologic studies depression scale.

a Unless indicated otherwise.

b Defined as requiring assistance with at least one basic ADL.

c Defined as whether injured seriously enough to need medical treatment.

d Defined as engaging in moderate or vigorous physical activity once a week or less or engaging only in mild activity.

e The proportional discrepancy between chronological age and felt age, where higher scores indicate feeling younger than one's actual age.

**Table 2:** Results of the logistic regression model predicting low gait speed (< 0.8 m/s) at follow-up.

	Unadjusted^a^			Adjusted^b^	
	*p*	Odds ratio [95% CI]		*p*	Odds ratio [95% CI]
Aging mindset predictors					
Optimism	0.191	0.922 [0.817, 1.041]		0.450	1.060 [0.911, 1.234]
Self-perceptions	**< 0.001**	0.575 [0.513, 0.645]		**< 0.001**	0.729 [0.627, 0.849]
Subjective age	0.315	1.427 [0.714, 2.854]		0.313	1.556 [0.660, 3.670]
Control variables					
Age in years				**< 0.001**	1.074 [1.041, 1.109]
Gender (reference = male)				0.782	1.063 [0.690, 1.638]
Depression (CES-D)				0.346	0.922 [0.779, 1.092]
Self-reported health (reference = good to excellent)				0.173	1.399 [0.863, 2.268]
Injurious fall between baseline and follow-up (reference = none)				0.772	0.929 [0.565, 1.529]
Fast gait speed (reference = slow)				**< 0.001**	0.088 [0.057, 0.135]
Physical inactivity (reference = active)				**0.046**	1.599 [1.008, 2.538]
ADL-dependence (reference = independent)				0.479	1.232 [0.692, 2.196]

a Nagelkerke *R*^2^ = 0.230; χ2(3) = 128.46, *p* < 0.001.

b Nagelkerke *R*^2^ = 0.541; χ2(11) = 350.87, *p* < 0.00.

**Table 3:** Results of the logistic regression model predicting ADL dependence at follow-up.

	Unadjusted^a^			Adjusted^b^	
	*p*	Odds ratio [95% CI]		*p*	Odds ratio [95% CI]
Aging mindset predictors					
Optimism	0.514	0.953 [0.823, 1.102]		0.516	0.948 [0.806, 1.114]
Self-perceptions	**< 0.001**	0.518 [0.447, 0.601]		**< 0.001**	0.667 [0.561, 0.792]
Subjective age	0.851	1.086 [0.457, 2.582]		0.289	1.642 [0.656, 4.109]
Control variables					
Age in years				0.984	1.000 [0.967, 1.035]
Gender (reference = male)				0.912	0.973 [0.605, 1.567]
Depression (CES-D)				0.291	1.096 [0.924, 1.300]
Self-reported health (reference = good to excellent)				**0.002**	2.752 [1.459, 5.189]
Injurious fall between baseline and follow-up (reference = none)				0.784	1.077 [0.632, 1.837]
Fast gait speed (reference = slow)				**< 0.001**	0.386 [0.231, 0.644]
Physical inactivity (reference = active)				0.250	0.736 [0.437, 1.240]
ADL-disability (reference = independent)				**< 0.001**	5.249 [3.130, 8.801]

a Nagelkerke *R*^2^ = 0.242; χ2(3) = 112.42, *p* < 0.001.

b Nagelkerke *R*^2^ = 0.388; χ2(11) = 190.53, *p* < 0.001.

**Table 4:** Results of the logistic regression model predicting physical inactivity at follow-up.

	Unadjusted^a^			Adjusted^b^	
	*p*	Odds ratio [95% CI]		*p*	Odds ratio [95% CI]
Aging mindset predictors					
Optimism	**< 0.001**	0.809 [0.719, 0.909]		0.050	0.880 [0.774, 1.000]
Self-perceptions	**< 0.001**	0.676 [0.609, 0.751]		**< 0.001**	0.795 [0.700, 0.904]
Subjective age	0.823	1.080 [0.550, 2.121]		0.519	1.275 [0.609, 2.673]
Control variables					
Age in years				0.188	1.018 [0.991, 1.046]
Gender (reference = male)				0.565	1.114 [0.772, 1.607]
Depression (CES-D)				0.106	0.888 [0.769, 1.025]
Self-reported health (reference = good to excellent)				0.117	1.382 [0.922, 2.072]
Injurious fall between baseline and follow-up (reference = none)				0.460	1.174 [0.767, 1.798]
Fast gait speed (reference = slow)				**< 0.001**	0.492 [0.335, 0.720]
Physical inactivity (reference = active)				**< 0.001**	3.454 [2.350, 5.077]
ADL-disability (reference = no disability)				0.988	0.996 [0.602, 1.649]

a Nagelkerke *R*^2^ = 0.176; χ2(3) = 96.74, *p* < 0.001.

b Nagelkerke *R*^2^ = 0.324; χ2(11) = 189.63, *p* < 0.001.

## Summary

- Key points
  ○ This study provides the first evidence that older individuals who had more positive self-perceptions of aging at baseline were better protected against negative physical consequences following a subsequent fall.
  ○ These results remained statistically significant after adjusting for important covariates including base-line gait speed, ADL dependence and physical inactivity, identifying self-perceptions of aging as an important independent predictor of physical recovery following a fall.
  ○ These novel findings highlight the potential of psycho-social factors to influence physical recovery from a fall in older adults.
- Why does this paper matter?
  ○ The present findings identify self-perceptions of aging as a robust predictor of physical function and disability following a fall, independent of other important factors such as age, gender, and pre-fall physical function.
  ○ These novel observations advance our understanding of the psychological factors impacting physical recovery from a fall and suggest that assessment of self-perceptions of aging could help identify older adults requiring additional support following a fall.
  ○ This could lead to enhanced recovery outcomes for older adults, ultimately reducing the burden of disability and improving quality of life after falls.
